# Catalytic Pyrolysis of Sinapic Acid on Nanoceria: Surface Complexes, Valorization of Products, Experimental, and Atomistic Approaches

**DOI:** 10.1002/cssc.202501249

**Published:** 2025-12-12

**Authors:** Tetiana Kulik, Nataliia Nastasiienko, Borys Palianytsia, Max Quayle, Andriiy Nastasiienko, Philip R. Davies, Duncan F. Wass, Alberto Roldan

**Affiliations:** ^1^ Cardiff Catalysis Institute Cardiff University Cardiff UK; ^2^ Chuiko Institute of Surface Chemistry NAS of Ukraine Kyiv Ukraine

**Keywords:** canolol, decarboxylation, experimental kinetic data, lignin‐rich biomass conversion, transition state

## Abstract

In this work, we investigated the pyrolysis of sinapic acid (SA) as a lignin S‐units model compound on the nanoceria catalyst. We employed various techniques to unravel the pyrolysis mechanism, including temperature‐programmed desorption mass spectrometry, thermogravimetric, and IR spectroscopic techniques, complemented with atomistic simulations. From spectroscopic data and atomistic models, we report that SA interacts with the catalyst via its carboxyl group and aromatic functional groups; the amounts of various surface complexes depend on the acid concentration. Conformational analysis revealed that parallel adsorption on ceria was preferred over the perpendicular one (Δ*E*_0_ = −154 kJ mol^−1^). The main pyrolysis products are associated with transformations of phenolate complexes, with the predominant formation of syringol and with decarboxylation of carboxylates, forming 4‐vinyl syringol, well known as canolol, thanks to its exceptional antioxidant properties. Modeling the transition state between the SA and its vinyl analog, canolol, displayed an additional intramolecular decarboxylation pathway with an activation energy barrier of +189 kJ mol^−1^. This is consistent with the activation energy *E* ^≠^ = 194 kJ mol^−1^ calculated from experimental kinetic data, and complements other established decarboxylation pathways. Methyl‐syringol, cresol, phenol, toluene, benzene, and other aromatics were found among the catalytic pyrolysis products of SA.

## Graphical abstract

Sinapic acid interacts with nanoceria through its carboxyl and aromatic groups, forming parallel and perpendicular surface complexes whose orientation depends on acid concentration and governs the kinetics and activation energy of forming valuable pyrolysis products—canolol, phenols, and other platform aromatics. These insights are crucial for optimizing thermochemical conversion of S‐type lignin‐rich biomass into bio‐oil and desired chemicals.

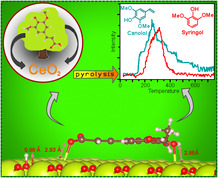

## 1 Introduction

Lignin an aromatic component of lignocellulose can be used as a renewable source of valuable chemicals. This biopolymer is found in plant cell walls, providing structural and protective functions [1]. Lignin macromolecules consist of phenylpropanoid lignin units (p‐hydroxyphenyl, H; guaiacyl, G; and syringyl, S‐units). They are connected by both ether and carbon–carbon linkages in different configurations. The configuration and quantitative content of these H–, G–, and S–units depend on the species of plant and the conditions of its cultivation [1, 2, 3].

Lignin is the second‐most‐common natural polymer worldwide [4]. Today, lignin is a by‐product in pulp and paper production, the agro–industrial complex, food and wood processing, biofuel production, and other industries [5, 6, 7, 8, 9, 10], with the lignin volume obtained only from pulp and paper enterprises reaching 50–70 million tonnes [7]. The production of biofuels is expected to increase in the future, leading to an increase in lignin co‐production [8]. Although most of this biopolymer is currently combusted [7], it has excellent potential as an environmentally friendly and renewable source of valuable chemicals and materials [9], and it expects lignin and its derivatives are expected to play a crucial role in alleviating society's dependence on fossil fuels to produce bulk chemicals [10]. However, continuous development of processes is necessary to ensure biorefineries’ long‐term stability and viability [4]. Therefore, searching for cost‐effective lignin valorization technologies is attractive but challenging for modern science [11].

Various pyrolysis methods are the most promising for developing technologies to efficiently convert nonfood lignin‐rich biomass into valuable chemical products and biofuels [5, 6, 12, 13, 14, 15, 16, 17, 18]. This is due to their environmental friendliness, low cost, and ability to effectively break down this biopolymer's rigid structure. A challenge for pyrolysis is the large number of products formed simultaneously, which have varying values as products and must be separated. Using catalysts enables increased processing selectivity and reduce energy costs [17, 18].

Ceria (CeO_2_) is an effective redox catalyst for biomass pyrolysis [19]. Cerium surface atoms can change the oxidation state from Ce^4+^ to Ce^3+^ and vice versa; simultaneously, ceria's surface releases or accumulates oxygen atoms [20]. Introducing oxygen vacancies and accompanying Ce^3+^ ions leads to a distortion of the local symmetry, particularly causing a change in the length of the Ce—O bonds. As the size of a CeO_2_ crystal decreases, the number of oxygen defects typically increases, which is a key factor in maximizing catalytic activity [21].

It has been shown that CeO_2_ catalytically converts vapors from the pyrolysis of rapeseed straw, poplar, cypress, and bagasse to ketones at temperatures below 400°C, with conversion rates of up to 34% [22]. CeO_2_ can further be combined with other oxides to enhance its catalytic properties [23, 24, 25]. We have previously investigated the catalytic pyrolysis of caffeic acid, cinnamic acid, vanillic acid, ferulic acid, catechol, and guaiacol [26, 27, 28]. These compounds to serve as model compounds for H– and G–units, which are widely distributed in the lignins of coniferous trees and grasses [29, 30]. Moreover, lignins usually incorporate high‐value molecules, such as various polyphenols, flavonoids, and p‐hydroxycinnamic acids, into their macromolecular structure along with their phenylpropanoid H–, G–, and S–units [30, 31]. Based on our previous spectroscopic techniques data, it was concluded that the formation of 4‐vinyl catechol, and 4‐vinyl guaiacol resulted from the decomposition of various carboxylate complexes of corresponding p‐hydroxycinnamic acids (caffeic and ferulic acids) on the ceria surface [26, 28]. At the same time, guaiacol, catechol, and phenol were formed due to the breakdown of the complexes formed by the functional groups of the aromatic core of p‐hydroxycinnamic acids on the ceria surface.

Sinapic acid (SA), 3,5‐dimethoxy‐4‐hydroxycinnamic acid, shares many structural similarities with S‐units of lignin and is often incorporated as an integral part of its structure [30]. Moreover, SA and its derivatives are found in significant quantities in various plants (vegetables, oilseed crops, fruits, and cereals) [32]. Rapeseed, in particular, is noted for one of the highest contents of SA and its derivatives [32, 33]. Given that rapeseed is one of the three oilseed crops with the most significant cultivation volumes [33], the waste of rapeseed oil production is a renewable source of SA and such a valuable product of its decarboxylation as 4‐vinyl syringol known as canolol. Canolol is a product with high added value [32, 33]. It can be used in various applications (pharmaceutical, cosmetology, polymer, and food industries) primarily due to its powerful antioxidant properties and broad bioactivity [32].

Therefore, the study of SA catalytic pyrolysis can provide a deeper understanding of the pathways for desirable product formation, and also mechanisms of thermal transformations of S‐type lignin, which predominates in hardwood biomass. Consequently, in this work, we investigated the pyrolysis of SA as a model compound of S‐units lignin on the CeO_2_ catalyst. Temperature‐programmed desorption mass spectrometry (TPD MS), thermogravimetric analysis, Fourier transform‐infrared (FT‐IR) spectroscopy, and atomistic modeling have been used to elucidate the structure of surface complexes of SA, mechanisms, and products of their thermal transformations, including decarboxylation with the high‐value‐added product, canolol, formation.

## 2 Results and Discussion

### 2.1 FT‐IR Spectroscopy Studies

**FIGURE 1:**
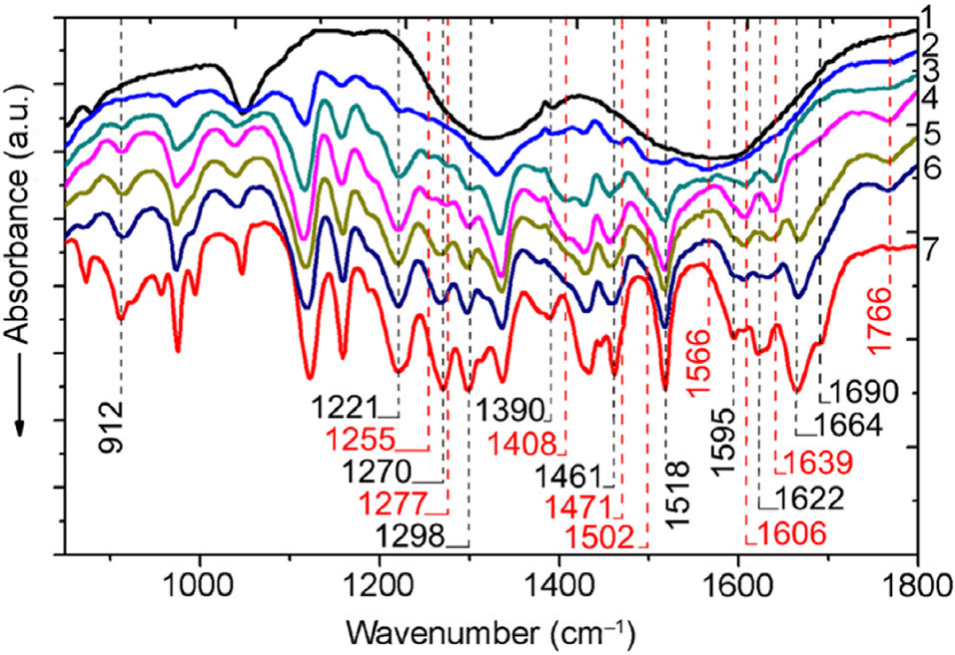
FT‐IR spectra of pure CeO_2_ samples (1) and SA/CeO_2_ samples with different concentrations of SA (0.1, 0.3, 0.6, 0.9, and 1.2 mmol/g, respectively 2−6) and pure SA (7).

**FIGURE 2:**
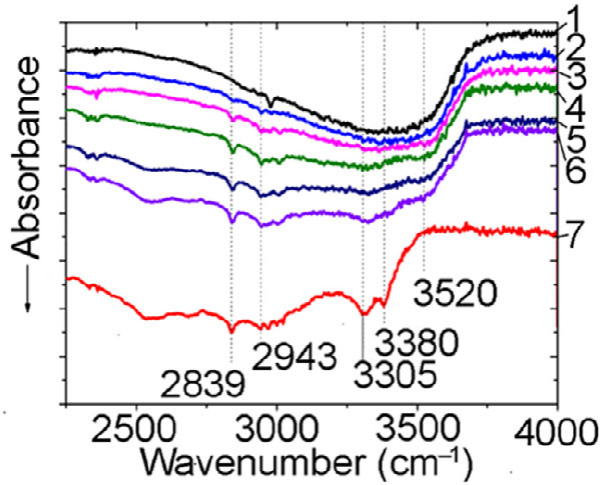
FT‐IR spectra of pure CeO_2_ samples (1) and samples SA/CeO_2_ with different concentrations of SA (0.1, 0.3, 0.6, 0.9, and 1.2 mmol/g, respectively 2−6) and pure SA (7).

FT‐IR spectroscopy is a powerful tool in studying molecular structure, allowing direct appreciation of the interaction between adsorbed molecules and the catalyst. It was employed on samples with different amounts of SA on the CeO_2_ surface (0.1−1.2 mmol) to establish the functional groups driving the interaction with the surface (Figures 1 and 2). We use the standard notation to describe stretching (*ν*) and deformation (δ) vibrational modes. Symmetric (s) and asymmetric (as) vibrations are denoted as subscripts.

As shown in Figure 1, the *ν*(C=O) bands of SA dimers at 1664 and 1690 cm^−^^1^ became almost invisible upon low SA loading from 0.1 to 0.3 mmol/g. The peak at 3076 cm^−1^, attributed to the carboxyl group *ν*(OH) [33, 34, 35, 36], practically disappears in the SA concentration range of 0.1–0.6 mmol/g, Figure 2. Similarly, the δ(ОН) intensity of the carboxyl group at 912 cm^−1^ decreased significantly [36]. Changes in the absorptions of the aromatic ring were also found. Instead of the *ν*(C=C)_ar_ bands at 1595 and *ν*(C=C) at 1622 cm^−1^, a broad band at 1605 cm^−1^ appeared. At high loading, i.e., 0.6–1.2 mmol/g, bands in the 2400–2700 cm^−1^ region and several other bands of pure SA, especially C=O bands of cyclic and linear dimers at 1664 and 1690 cm^−1^, occurred [36].

The apparition of new bands upon SA adsorption indicates the formation of surface complexes with the participation of the carboxyl group. In particular, two new bands at 1390 and 1408 cm^−1^ are associated with the stretching vibration of *ν*(С—О), whereas the other new bands at 1766 and 1639 cm^−1^ are assigned to the stretching vibration of *ν*(C=O). These four bands could correspond to weakly‐ or hydrogen‐bonded and monodentate complexes formed through the COOH group. Also, other bands appear that could be associated with the formation of carboxylate complexes: *ν*_s_(СОО^–^) at around 1470 and *ν*_as_(СОО^–^) at 1566 and 1502 cm^−1^.

The structure of surface carboxylate complexes on metal oxide catalysts, including those based on ceria, was successfully identified in our previous work [25] by analyzing the ‘Δ*ν*’ value of the separation between asymmetric and symmetric carboxylate stretches Δ*ν *= *ν*_as_(COO^−^) − *ν*_s_(COO^−^) or, in the case of monodentate coordination, between C=O and C=O stretches (Δ*ν *= *ν*(C=O) − *ν*(CO)) [37]. The analysis of the magnitude of ‘Δ*ν*’ allows the conclusion that there are several types of binding: monodentate complexes with Δ*ν *= *ν*(C=O) − *ν*(CO) = 1639–1408 = 231 cm^−1^; bidentate chelate carboxylates with Δ*ν *= *ν*_as_(COO^−^) − *ν*_s_(COO^−^) = 1566–1470 = 96 cm^−1^; weakly bonded complexes or hydrogen‐bonded complexes with Δ*ν *= *ν*(C=O) − *ν*(CO) = 1766–1390 = 376 cm^−1^. The band at 1766 cm^−1^ was assigned to the stretching vibration of the C=O group with hydrogen bonding or other weak bonding based on the analysis of the stretching vibration of this group for the monomer of the SA molecule in the gas phase. The monomer of SA has a frequency of about 1800 cm^−1^, which is in accordance with the experimental spectrum from the NIST [38], which will be presented below in the density functional theory (DFT) section. The interaction of the C=O group of the acid with the formation of different types of bonds, for example, hydrogen bonds in dimers or with water molecules [36], as well as in monodentate and bidentate complexes with active sites, will shift the absorption frequency toward shorter return wavelengths. This shift will be more significant according to the greater strength of the bond [36].

Intense absorptions at 1270 and 1298 cm^−1^ for SA are associated with C—O—H vibrations of phenolic and carboxyl OH groups [34, 35, 39]. For the SA/СеО_2_ samples, the intensity of both bands is significantly lower, especially for concentrations of 0.1–0.6 mmol/g. In addition, instead of a band at 1270 cm^−1^ associated with C—O—H, two maxima rise at 1255 and 1277 cm^−1^. This splitting is explained by SA interacting very efficiently with the CeO_2_ surface via the phenolic hydroxyl group, as confirmed by DFT modeling; see discussion below. This splitting disappears via overlapping with the intense band at 1270 cm^−1^ at the SA concentrations above 0.9 mmol/g. Also, FT‐IR spectroscopic data indicate the possibility of SA binding to the oxide through the methoxy group because the absorption at 1221 cm^−1^ (*ν*_as_(С—О—СН_3_)_аr_) and at 1461 cm^−1^ (δ(СН_3_)) have a significantly lower intensity in the SA/СеО_2_ spectra (0.1–0.6 mmol/g).

In the spectra of SA/CeO_2_, there are changes in the absorptions of the OH_ar_ groups, Figure 2, suggesting that these groups may also be involved in the interaction with the oxide. The peaks at 3320 and 3380 cm^−1^ correspond to *ν*(OH)_ar_ vibrations associated with the formation of SA intermolecular hydrogen bonds. However, for SA/СеО_2_, their intensity decreases, and a broad shoulder appears at around 3500 cm^−1^. Absorption of valence vibrations of phenolic groups of dimers, which are connected by an internal molecular bond and free phenolic hydroxyls, may appear in this frequency range. This is due to the destruction of intermolecular hydrogen bonds during adsorption on CeO_2_. In addition, the interaction of methoxy groups with the oxide surface can destroy these groups’ internal hydrogen bonds with neighboring phenolic groups. In the IR spectrum, this is manifested by the appearance of vibrations in the region of free phenolic groups.

### 2.2 DFT Calculations

**FIGURE 3:**
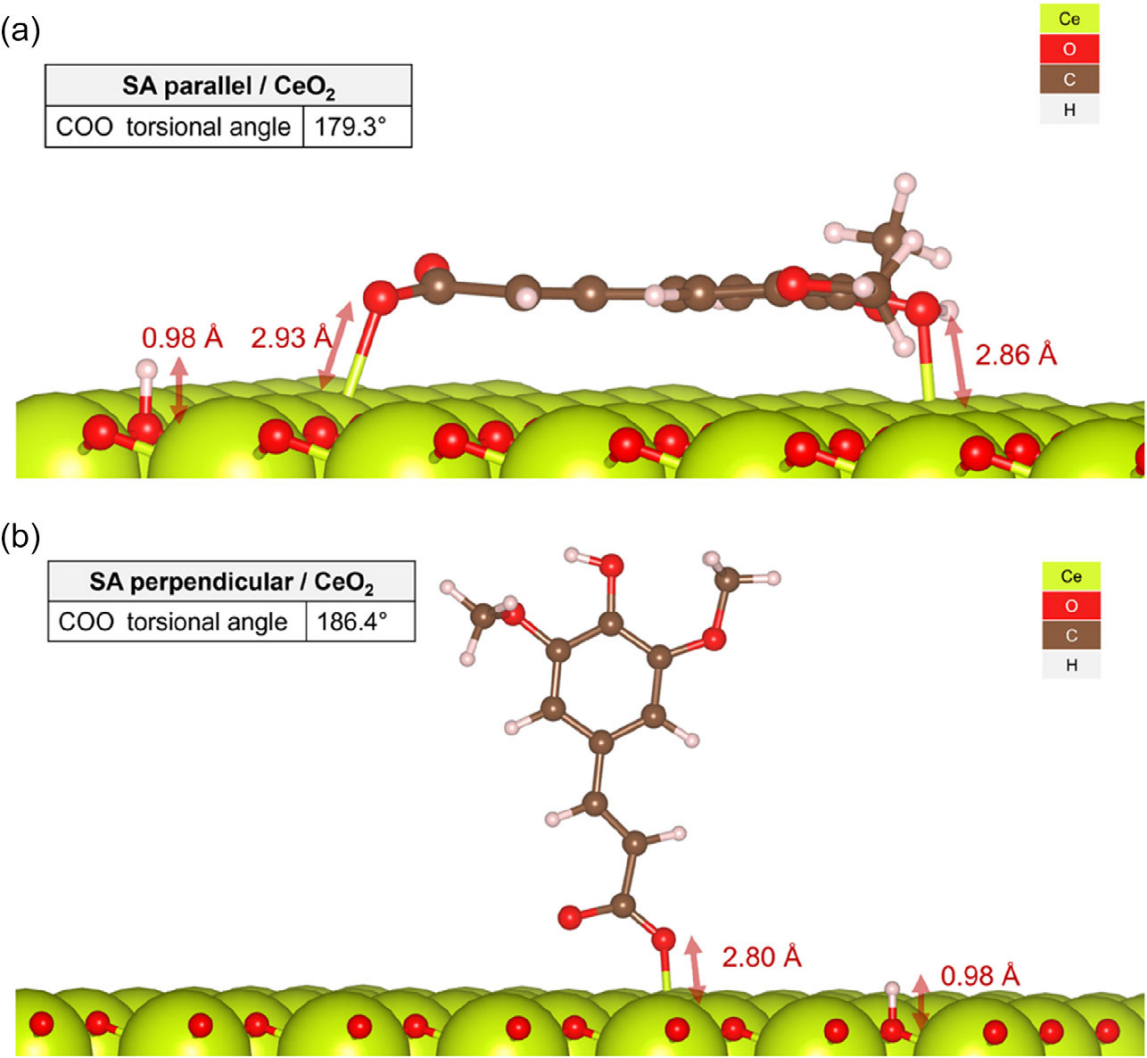
Dissociated SA over CeO_2_(111) in the parallel (a) and perpendicular (b) conformations. Distances between adsorbate atoms and surface atoms were measured relative to the *z*‐axis of the surface.

SA can adopt various positions on the CeO_2_(111) surface. Following previous modeling work on heterogeneous catalysis [40, 41, 42, 43, 44], two binding conformations were tested and the molecular and surface interatomic forces fully relaxed. These conformations were (1) the molecule lies parallel to the surface and binds to the catalyst through its aromatic ring system and (2) the molecule adopts a perpendicular conformation and binds to a Ce atom through its COO^−^ group upon the dissociation of the SA's acidic proton (Figure 3). Following the observations of previous studies of hydrogen clustering on CeO_2_(111) [45], the acidic hydrogen was placed on an O top site. Conformational analysis showed that the parallel conformation is energetically preferred (Δ*E*_0_ = −154 kJ mol^−1^), Table 1. The exothermic adsorption of SA on CeO_2_ (Table 1) suggests an increment of surface coverage with the concentration and the random stacking of complexes leading to partial bonding with the surface (monodentate) or even conformations stabilized by long–range interaction (H‐bonded complexes). The surface's redox properties, combined with the intense adsorption energy, may have positive implications for the pyrolytic activity.

**TABLE 1:** Summary of crucial interatomic distances, angles, and SA adsorption energy on the pristine CeO_2_(111) derived from atomistic simulations.

PBE‐D3	Parallel conformation	Perpendicular conformation
d(Ce–O_ACID_), Å	3.18	2.80
d(O‐H_TOP_), Å	0.98	0.98
COO torsional angle, °	179.3	186.4
Adsorption energy, kJ mol^−1^	−237.4	−83.0

**FIGURE 4:**
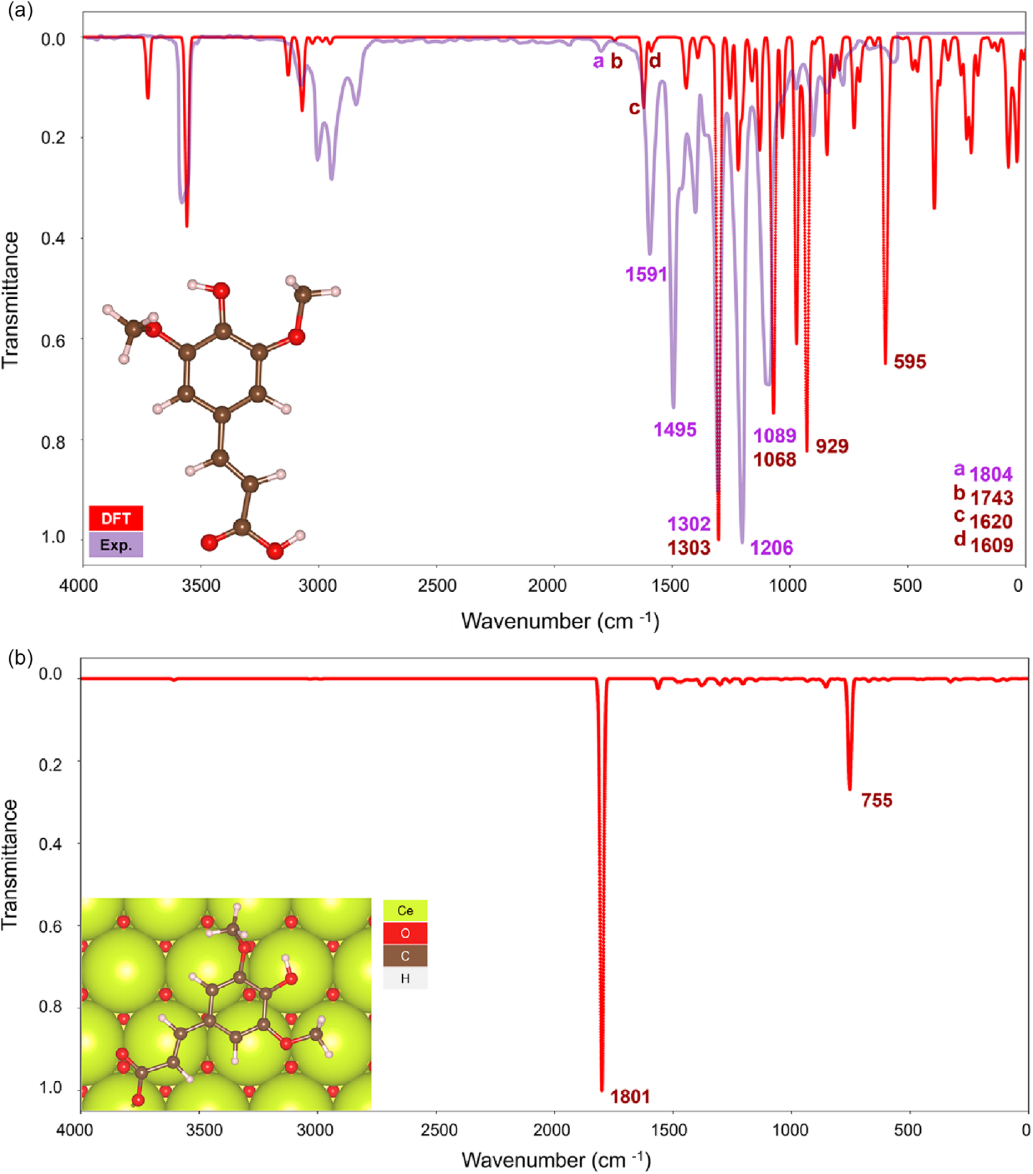
Simulated (red) and experimental (lilac) IR spectra of SA in the gas phase (a). Simulated IR spectra of SA conjugate base in the parallel conformation over CeO_2_(111) (b). Optimized structures are given in the insets. Experimental IR data taken from the National Institute of Standards and Technology [38].

The simulated IR spectrum for SA in the gas phase (Figure 4) showed satisfactory agreement with the overlayed experimental IR. While the model underestimated the intensities of the vibrational modes at 1495 and 1206 cm^−1^ and slightly overestimated the wavenumbers of the bands in the 2800–3100 cm^−1^ range, all key peaks were correctly identified, with the alkene C=C symmetrical stretch at 1302 cm^−1^ captured almost exactly.

However, the parallel SA/CeO_2_ IR spectrum presents a sharp symmetrical carboxylate *ν*(C—O) peak at 1801 cm^−1^ that dwarfs the rest of the spectrum compared to the experimental IR in Figure 1. Despite this, the model correctly predicted the presence of a peak corresponding to an alkene stretch at 1563 cm^−1^. The peak at 755 cm^−1^, the only other frequency with significant intensity, corresponded to an asymmetric methoxy stretch, which was found to be coupled with a carboxylate δ(C—O) mode.

**FIGURE 5:**
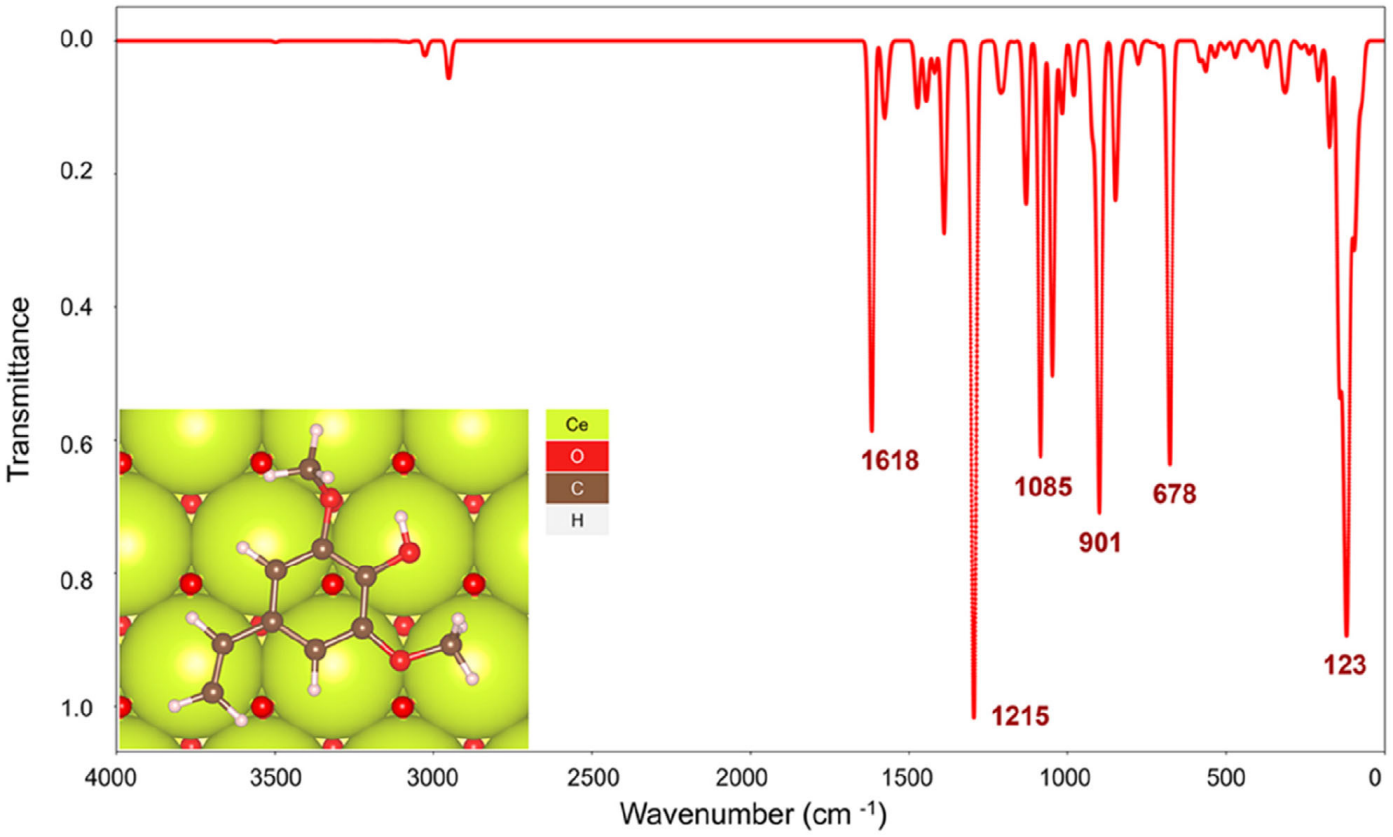
Simulated IR spectrum of 4‐vinyl syringol in the parallel conformation over CeO_2_(111). The optimized structure is given in the insets.

It is known that the pyrolysis of caffeic, ferulic, and coumaric acids on CeO_2_ was accompanied by the formation of vinyl pyrocatechol, vinyl guaiacol, and vinyl phenol, respectively [26, 27, 28]. Thus, atomistic simulations were conducted to shed light on the possibility of SA decarboxylation generating CO_2_ and 4‐vinyl syringol [46, 47]. The simulated IR spectrum of 4‐vinyl syringol over CeO_2_(111) (Figure 5) bears a close resemblance to the experimental spectrum, confirming the fast decarboxylation of SA on CeO_2_. The peak of highest intensity corresponds to an asymmetric methoxy stretch at 1215 cm^−1^.

**FIGURE 6:**
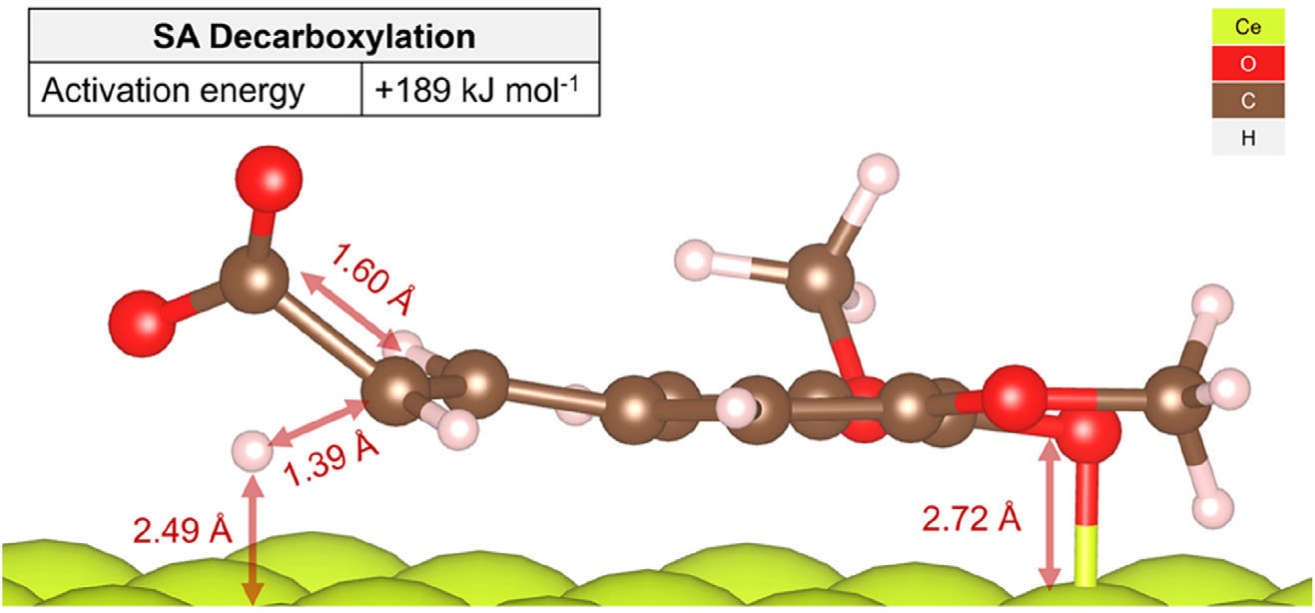
Representation of the transition state for the decarboxylation of SA over CeO_2_(111). The distances of the migratory hydrogen (2.49 Å) and the alcohol oxygen (2.72 Å) to the surface were measured with respect to the z‐coordinates of the topmost layer of Ce atoms.

Modeling the transition state between the SA and its vinyl analog revealed an intramolecular decarboxylation pathway with an activation energy barrier of +189 kJ mol^−1^ (Figure 6). The height of this barrier is well within the typical range for cyclic hydrocarbons [48] and would undoubtedly be surmountable under experimental pyrolysis conditions [49].

### 2.3 Pyrolysis of SA Over Ceria Catalyst

**FIGURE 7:**
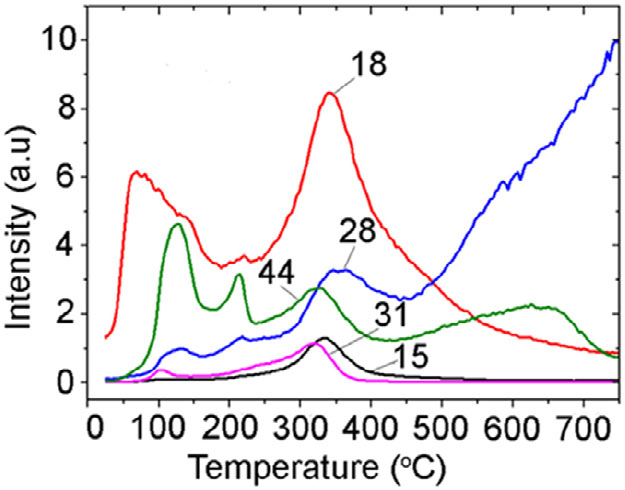
TPD curves obtained for ions with *m/z* 15 (CH_4_), 18 (H_2_O), 28 (CO), 31 (CH_3_OH), and 44 (CO_2_), during the pyrolysis of SA/CeO_2_ (0.6 mmol/g) sample.

According to the results of the TPD‐MS study, the thermal decomposition of SA on the СeO_2_ surface occurred in a temperature between 100°C and 450°C (Figure 7). In the process of pyrolysis for all SA/CeO_2_ samples, an intensive release of gaseous products was registered: СН_4_ (*m/z* 15, 16), (Н_2_О) (*m/z* 18), СО, С_2_Н_4_ (*m/z* 28), СН_3_ОН (*m/z* 31, 32), and СО_2_ (*m/z* 44).

**FIGURE 8:**
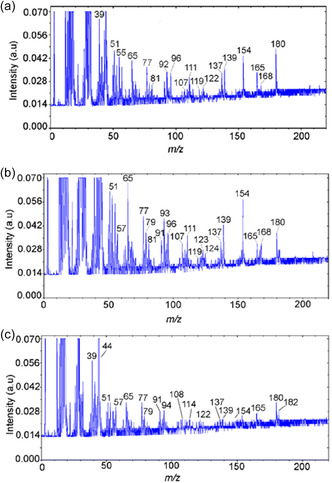
Mass spectra of pyrolysis products for the sample SA/CeO_2_ (1.2 mmol/g) obtained at temperatures of (a) 240°C, (b) 325°C, and (c) 430°C.

**FIGURE 9:**
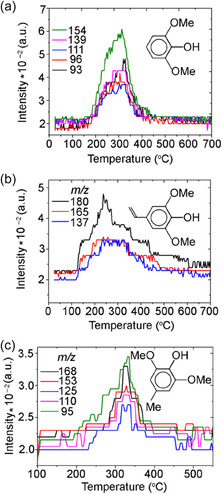
TPD curves for (a) molecular ion of syringol with *m/z* 154 and its fragment ions with *m/z* 139, 111, 96, 93; (b) molecular ion of canolol with *m/z* 180 and its fragment ions with *m/z* 165, 137; (c) molecular ion of methyl‐syringol with *m/z* 168 and its fragment ions with *m/z* 153, 125, 110, 95; obtained during the pyrolysis of the SA/CeO_2_ sample (1.2 mmol/g).

**SCHEME 1:**
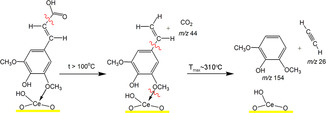
A suggested mechanism for SA decarboxylation leading to syringol over ceria. Note that the perpendicular representation of syringol enhances the sketch's clarity.

A large number of ion signals were detected in the mass spectra from 180°C to 450°C for high SA concentrations (0.6−1.2 mmol/g) (Figure 8). This result contrasts with the observations during the pyrolysis of ferulic and vanillic acids [27]. The main products of SA pyrolysis on the CeO_2_ surface are presented in Table 2. For concentrations of 0.6−1.2 mmol/g, a molecular ion of syringol with *m*/*z* 154 and its fragment ions with *m/z* 139, 111, 93, 96 were registered in the mass spectra of pyrolysis products (Figure 8a–c). That is in good agreement with the literature [38] and corresponds to syringol (*m*/*z* 154 (100%), 139 (46%), 111 (22%), 96 (17%), 93 (18%)). Syringol formation was observed in a wide temperature range, with *T*_max_ ≈ 320°C (Figure 9a, Table 2). It is similar to pyrocatechol and guaiacol, which are formed during the pyrolysis of caffeic and ferulic acids over ceria and alumina [27, 28, 50]. Therefore, we suggest that syringol formation (Scheme 1) occurs via the thermal decomposition of SA complexes bound to the surface via their methoxy groups, as shown in the atomistic models (Figures 3 and 6).

**TABLE 2:** SA catalytic pyrolysis over ceria: products, temperature range of product release *T*_range_, temperature of the maximum desorption rate *T*_max_, and calculated activation energy *E* ^*≠*^.

Product	Ion fragments, *m/z*	*T*_max_ */T*_range,_ °C	*E* ^*≠*^, kJ mol^−1^ (*ν*_0_ = 10^13^ s^−1^)^a^	*E* ^*≠*^, kJ mol^−1^ a	References
Dehydration					
H_2_O	18	~80–120	102–113	—	—
		~340	177	—	—
Decarboxylation					
CO_2_	44	~130	116	—	—
		~214	140	—	—
		~324	172	—	—
		~450–500	—	—	—
		~650	266	—	—
Demethoxylation					
CH_3_OH	31/32	~100	107	—	—
		~218	142	—	—
		~320	171	—	—
Demethylation					
CH_4_	15/16	~338	176	—	—
		~300	165	—	—
Decarboxylation of carboxylate complexes					
4‐Vinyl syringol	180/165/137	~150	123	87*	38, 51
Canolol		~240	148	106*	
		~330	174	124*	
		~400	194	139*	
Decomposition of phenolate complexes					
Syringol	154/139/111/93/96	~310	168	97**	38
4‐Methyl syringol	168/153/125	~325	172	99**	51
Aromatics release					
Benzene	78/77	300–380	—	—	38
Toluene	91/92	~325	172	123*	38
Cresol	107/108	~340	177	126*	38
Pyrocatechol	110/64	~325	172	—	38
4‐Ethyl syringol	182/167	230−550	—	—	51

a Activation energy *E* ^*≠*^ was calculated approximately by using equation *Е* ^*#*^ = ln(*B*/ln *B*) *RT*_max_ for reaction order *n* = 1, and pre‐exponential factors *ν*_0_ = 10^13^,

* *ν*_0_ = 10^8^, and

** *ν*_0_ = 10^6^ s^−1^ [5, 25, 26, 50, 56, 57].

**SCHEME 2:**
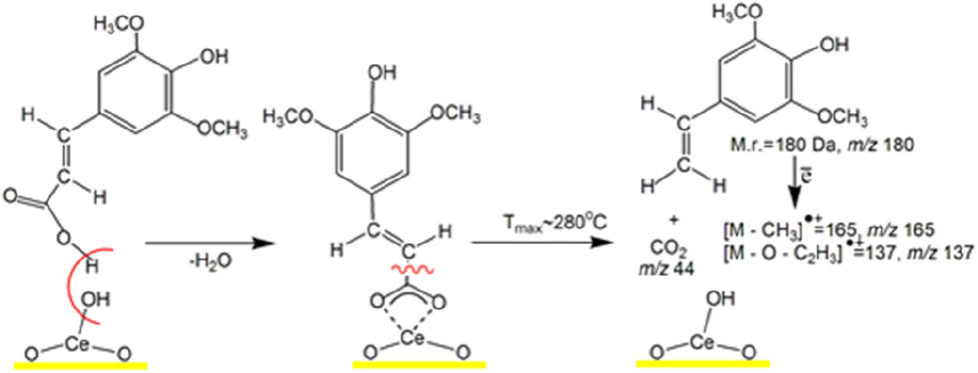
A possible mechanism for 4‐vinyl syringol (canolol) formation during the decomposition of the bidentate chelate carboxylate over ceria.

**SCHEME 3:**
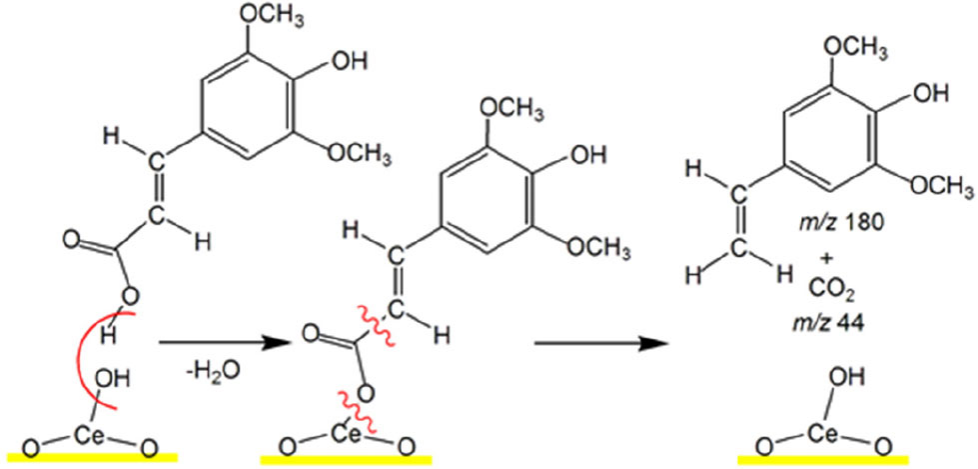
A possible mechanism for canolol formation during decomposition of the monodentate complexes over the ceria surface. Note that the perpendicular representation of syringol enhances the clarity of the sketch.

Formation of high‐value‐added product 4‐vinyl syringol, named canolol, occurs during pyrolysis of SA/CeO_2_ samples with high loading (0.9 and 1.2 mmol/g). That was confirmed by a molecular ion of 4‐vinyl syringol with *m/z* 180 and its fragment ions with *m/z* 165 and 137 in mass spectra of pyrolysis products (Figure 8a–c) and also by TPD curves for these ions (Figure 9b) [51, 52, 53]. The formation of canolol occurs due to the decarboxylation of several types of surface complexes (Schemes 2 and 3), which were confirmed by FT‐IR spectroscopy data and atomistic modeling above. The same complex decarboxylation processes were observed previously for the catalytic pyrolysis of ferulic, coumaric, and caffeic acids with the release of the corresponding 4‐vinyl phenols and CO_2_ [26–28, 50].

As shown in Figure 9b, the TPD curves of molecular and fragment ions of 4‐vinyl syringol exhibit a complex shape due to the superposition of multiple decarboxylation processes. Since it is known [37] that the bond strength in carboxylate complexes increases in the following order: weakly bonded and hydrogen‐bonded complexes < monodentate‐bonded complexes < bidentate chelate carboxylates. Therefore, at the lowest temperature, hydrogen‐bonded complexes will decompose (*T*_max_ ≈ 150°C), then monodentate (*T*_max_ ≈ 250°C), bidentate chelate (*T*_max_ ≈ 320°C), and at the highest temperature (*T*_max_ ≈ 400°C) decarboxylation of the phenolate complex with a parallel conformation will occur in agreement with the simulated activation energy of 189 kJ mol^−1^ (Figure 6). We hypothesize that the activation energy for decarboxylation would increase in the same order. The temperature of the maximum desorption rate, *T*_max_, was often employed for semi‐quantitative calculation of the activation energies of reactions [5, 25, 26, 54−57]. Calculating activation energies were conducted by approximated equation suggested by Kislyuk and Rozanov based on Redhead's approaches [56, 57]: *Е* ^*#*^ = ln(*B*/ln *B*) *RT*_max_ (*B *= (*n ν*_0_ *T*_max_ *C*_max_^*n* − 1^) *b*), where *n* is the reaction order, *ν*_0_ is the pre‐exponential factor, *C*_max_^*n*^ ^− 1^ is the concentration of the adsorbate at *T*_max_, and *b* is the value of the sample heating rate (*b* = 0.167 C s^−1^).

This approximate equation is often used to estimate the activation energy when the linear Arrhenius plot method cannot be applied due to the complex shape of the TPD peak or when several peaks overlap. We have previously used these approaches in our works [5, 25, 54, 55], which allowed us to obtain valid results. In the present work, we calculated the activation energies of the formation of the main products for first order (*n* = 1) using the so‐called “ideal” value of the pre‐exponential factor *ν*_0_ = 10^13^ s^−1^, when the reaction proceeds through a disordered transition state. In addition, we also used the pre‐exponential factor, which is characteristic of processes proceeding through highly ordered cyclic transition states. We used the values *ν*_0_ = 10^8^ and 10^6^ s^−1^ of the pre‐exponential factor obtained by using the Arrhenius plot method for the same processes of hydroxycinnamic acids on the surface of ceria and alumina [26, 28, 50]. The obtained data are presented in Table 2. In particular, the activation energy for decarboxylation of the complex with a parallel conformation calculated from the experimental data is 194 kJ mol^−1^, which is only 2.6% less than the theoretical value of 189 kJ mol^−1^ obtained by atomistic modeling.

The most extensive release of methylated product, 4‐methyl‐syringol, was observed in the 320−330°C temperature range with the SA/CeO_2_ loading of 0.6−1.2 mmol/g (Figures 8 and 9c). 4‐Methylsyringol (*m/z* 168, 153, 125) [13] could be formed on ceria as a result of various catalytic reactions, including possible transmethylation processes due to demethoxylation and others. That is confirmed by an intense peak of methanol desorption (*m/z* 32, 31) for the SA/CeO_2_ samples (0.9–1.2 mmol/g). Such a peak was registered at about 320°C (Figure 7). Since some methoxy groups can remain on the oxide surface [53, 58], a course of transmethylation reactions is possible. Methylated products were also observed during the pyrolysis of ferulic acid over ceria [26].

Comparing the results of pyrolysis of ferulic acid [26] and SA on the surface of CeO_2_, it is found that more products are formed in the latter case. The second methoxy group in the SA likely increases the product's permutation and its reactivity. In addition, the O—CH_3_ bond homolysis is the pyrolytic decomposition rate‐limiting stage of guaiacol and syringol [59], and, therefore, the reactivity of this functional group depends on its strength. The dissociation energies of these bonds in guaiacol and syringol are close, +285.3 and +283.7 kJ/mol, respectively, consistent with their reactivity [59].

### 2.4 Distribution of Different Types of SA Complexes on the CeO_2_ Surface Depending on Acid Concentrations

**FIGURE 10:**
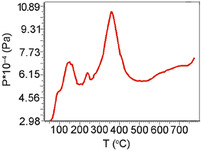
The Pressure of pyrolysis products at the temperature of the sample (*P*/*T* curve) obtained during the pyrolysis of the SA/CeO_2_ sample (1.2 mmol/g).

**FIGURE 11:**
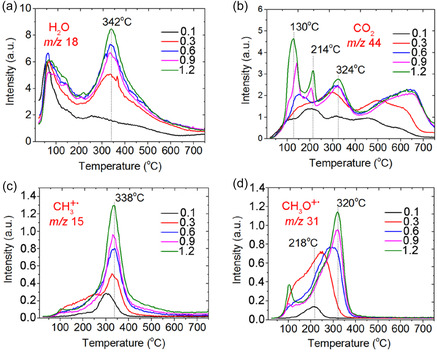
TPD curves for ions with *m/z* 18 (a), 44 (b), 15 (c), 31 (d) for samples of SA/СеО_2_ (0.1, 03, 0.6, 0.9, 1, 2 mmol/g).

Comparative analysis of TPD curves and the Pressure/Temperature (*P*/*T*) curves obtained during the pyrolysis of the SA/CeO_2_ can provide additional information and a complete picture of the desorption of gaseous products during this process (Figures 10 and 11). The most intensive gas evolution occurred in the 250−450°C temperature range (Figure 10). This effect is due to the fact that in this temperature range, the decomposition of the vast majority of carboxylate and phenolate complexes occurs, accompanied by the desorption of catalytic pyrolysis products.

Figure 11 shows the TPD curves of various gaseous products: H_2_O (*m/z* 18), CO_2_ (*m/z* 44), and CH_3_OH (*m/z* 31). Using Figure 11 allows us to compare the relative intensities of TPD peaks and assess the efficiency of various pyrolysis processes. The intensity of TPD peaks for these gaseous products is high for all SA/CeO_2_ samples. Moreover, their intensity increases with increasing SA concentration. From the analysis of the curves, we can obtain information about the structure of the SA surface layer. The most important results are the release of CO_2_ (*m/z* 44) and CH_3_OH (*m/z* 31), which are associated with decarboxylation and demethoxylation, respectively. Several TPD peaks are visible for the ion with *m/z* 44 (Figure 11b). The release of CO_2_ occurs during the decomposition of various SC complexes, including aromatic ones (Schemes 1–3).

Thus, the following stages can be proposed based on the TPD peaks at *m/z* 44, which are associated with the decomposition of (i) weak or hydrogen‐bonded complexes at 130°C, (ii) monodentate complexes (~214°C), and (iii) bidentate chelate and phenolate complexes (~323°C). This assignment is supported by the nature of the release of 4‐vinylsyringol and syringol, since the release of syringol is also accompanied by the release of CO_2_ (Scheme 1). In particular, the formation of 4‐vinylsyringol s (*m*/*z* 180) occurs over a wide temperature range from ~120 to ~550°C, as noted above, due to overlapping decarboxylation of different surface complexes, Figure 9b. For the sample with a high SA surface loading, 0.9–1.2 mmol/g, the number of weakly bound complexes, i.e. H‐bonded and monodentate bonds, increases significantly, which can also be seen from the FT‐IR spectroscopy data (Figure 2). In particular, the absorption bands of acid dimers (2400–2700 cm^−1^) are most intense for SA/CeO_2_ (1.2 mmol/g). This results in increased intensities of the low‐temperature peaks in the TPD curves for CO_2_ at ~100–130°C and ~200°C for these SA/CeO_2_ samples, explaining the higher intensities observed for other stages in the TPD curves.

In the SA/CeO_2_ sample with the lowest loading (0.1 mmol/g), the highest intensity of the TPD peak for CO_2_ is observed at *T*_max_ = 209°C, confirming the formation of predominantly monodentate‐bonded complexes at low loading. Evidence of these complexes can be seen in the corresponding bands in Figure 1. This may indicate changes in the structure of the surface layer of the SA (0.1–1.2 mmol/g), which is also accompanied by changes in the desorption of the demethoxylation product CH_3_OH (*m/z* 31) (Figure 11d). The maximum of the release of *m/z* 31 for lower SA concentrations shifts to 218°C. Analysis of TPD curves for *m/z* 31, 44, 180, and 154 (Figures 9 and 11b,d) indicates that the most intensive demethoxylation processes occur in the temperature range of 320°C, i.e., synchronously with the decomposition of carboxylate, phenolate, and methoxylate complexes, which occur with the formation of syringol (*m/z* 154) and 4‐vinyl syringol (*m/z* 180).

FT‐IR spectra (Figure 4) demonstrate changes in the structure of the surface layer with increasing SA concentration on the surface. In particular, for the SA/CeO_2_ sample (0.1 mmol/g), the *ν*_s_(СОО^–^) bands at 1471 and the *ν*_as_(СОО^–^) at 1566 cm^−1^ are observed, which is associated with the bidentate chelate coordination of carboxylate groups. The relative intensity of these bands decreases at higher SA concentrations, and signs of other complexes appear. It should be noted that the excess of the intensity of syringol emission (*m/z* 154) for the SA/CeO_2_ samples (0.6–1.2 mmol/g), compared to 4‐vinyl syringol (*m/z* 180), indicates that complexes bound through methoxy‐, hydroxy‐groups, and complexes in the parallel SA conformation prevail on the surface.

### 2.5 Differential Thermal Analysis (DTA)/Differential Thermogravimetric Analysis (DTG)/Thermogravimetric (TG) Study of the SA/CeO_2_ Sample

**FIGURE 12:**
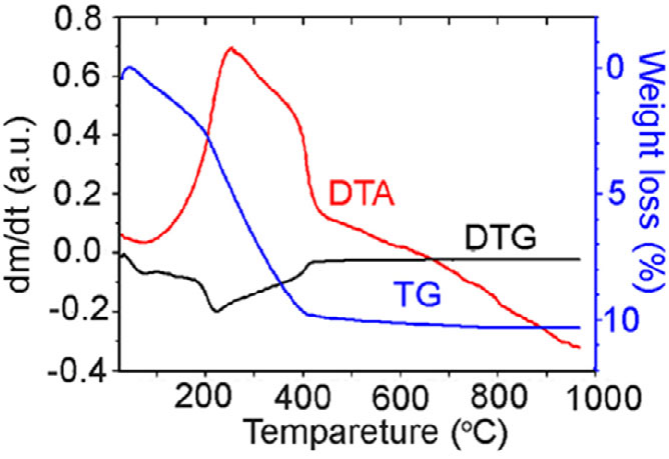
DTA, DTG, and TG curves for SA/СеО_2_.

The outputs from the DTA/DTG/TG study expose the acid's decomposition in the temperature range of 100–450°С (Figure 12). According to the DTG curve, several intensive processes coincide in the same temperature range of 200−400°С. The DTA/DTG/TG data analysis established that 87.8% of SA is converted into volatile products and 12.2% into coke.

Considering the first maximum on the DTG curve (Figure 12) at ~70°C, with the same on the TPD curve for *m*/*z* 18, shows that the first mass loss corresponds to releasing physically adsorbed water from the SA/СеО_2_ sample. The next maximum rate of mass loss on the DTG curve (at ~227, ~250, and ~400°C) corresponds to the decomposition of various surface complexes of SA together with oxidative destruction in the air atmosphere. Small loss of mass when heated above 400°С is probably due to the transformation of coke with the desorption of gaseous products. It can be seen from the DTA curve that the processes occurring up to 430°С are exothermic.

## 3 Conclusions

The pyrolysis of SA over ceria was studied using TPD‐MS, thermogravimetry, FT‐IR spectroscopy, and atomistic modeling techniques. Our experimental results and atomistic modeling showed that SA interacts with nanoceria via the carboxyl group and aromatic functional groups, forming complexes parallel and perpendicular to the catalyst surface. Moreover, the conformational analysis revealed that parallel adsorption on ceria was preferred over perpendicular adsorption (Δ*E*_0_ = −154 kJ/mol). It was shown that the formation of different surface carboxylates, e.g., bidentate chelate carboxylates, weakly‐bonded species, and monodentate‐bonded complexes, depends on the acid concentration on the surface.

The main products from the pyrolysis are associated with thermal transformations of phenolate complexes, with the predominant formation of syringol and also with decarboxylation, forming 4‐vinylsyringol, also known as canolol. Canolol is a product with high added value ($298 per 100 mg). Moreover, it has prospective market application value due to its powerful antioxidant properties and broad spectrum of bioactivity. Given the labor‐intensive, multistage, and expensive methods of its industrial production, developing alternative catalytic pyrolysis strategies for second‐generation nonfood biomass for canolol obtaining looks extremely promising. Importantly, modeling the transition state between the SA and its vinyl analog, canolol, displayed an additional intramolecular decarboxylation pathway with an activation energy barrier of +189 kJ/mol that is in excellent agreement with the calculated value of activation energy *E* ^≠^ = 194 kJ/mol from the obtained experimental kinetic data.

According to our data, the main compounds formed during the catalytic pyrolysis of SA were mainly canolol, syringol, methylated syringol, cresol, toluene, benzene, and phenol. The main gaseous products released during pyrolysis were H_2_O, CO, CO_2_, and methanol.

Comparing the noncatalytic pyrolysis of syringol and the catalytic pyrolysis of SA over ceria, it was found that using ceria can contribute to a significant decrease in the decomposition temperature of the lignin S‐units. We hope that this work provides a solid foundation for further investigations into the pyrolytic conversion of hardwood S‐type lignin–derived feedstocks into valuable products, including canolol, phenols, aromatics, substituted styrenes, and advanced biofuels.

## 4 Experimental

### 4.1 Materials

Nanosized ceria (99.5%, *S*_Ar_ = 71 m^2^/g, Alfa Aesar) and SA (≥98%, Sigma–Aldrich, St. Louis, USA) were used in this study. The CeO_2_ was pre‐calcined at 500°C for 2 h to remove organic matter. A series of SA/CeO_2_ samples (0.1, 0.3, 0.6, 0.9, and 1.2 mmol/g) was prepared by impregnating CeO_2_ with an ethanolic solution of SA. The resulting suspensions were stirred and air‐dried at room temperature.

### 4.2 FT‐IR Spectroscopy

In situ FT‐IR spectroscopy was recorded on a Thermo Nicolet Nexus Fourier transform IR spectrometer (Thermo Nicolet Corporation, Madison, WI, USA), using a Nexus Smart Collector in diffuse reflection mode over the range of 4000–400 cm^−1^. The resolution was ±4 cm^−1^, the total number of scans was 50, and the scan velocity was 0.5 cm/s. Before any FT‐IR spectra were taken, the CeO_2_ and SA/CeO_2_ samples were mixed with KBr (≥99%, Alfarus, Kyiv, Ukraine) in a 1:10 ratio. Pure SA was mixed with KBr at a ratio of 1:100. KBr was pre‐calcined at 500°C for 2 h. The IR spectra of SA/CeO_2_ samples were obtained and presented in this work.

### 4.3 TPD MS

TPD MS was performed using an MX‐7304 monopole mass spectrometer (Electron, Sumy, Ukraine) equipped with electron ionization and modified for pyrolysis kinetic studies [25, 54−56]. At the start of each experiment, a sample weighing 15 mg was placed in a quartz–molybdenum ampoule and pumped out at room temperature to a pressure of ~5 × 10^–5^ Pa. Heating was increased from room temperature to 750°C at a programmed linear rate of 0.17°C/s. Volatile pyrolysis products entered the ionization chamber of the mass spectrometer and were ionized and fragmented under the action of electrons. The total number of mass spectra recorded during the experiment reached ~240.

### 4.4 Thermogravimetric Analysis

Thermogravimetric analysis was performed using a TGA/DTA analyzer (Q‐1500D, Budapest, Hungary). Samples weighing 100 mg were heated from room temperature to 1000°C. The heating rate was 10°C/min in an air atmosphere.

### 4.5 DFT Calculations

DFT calculations were performed in the Vienna Ab Initio Simulations Package (VASP) [60, 61]. All calculations were carried out under the constraints of the generalized gradient approximation and long‐range dispersion corrections at the PBE‐D3 level [62, 63]. The Brillouin zone was sampled with a 3 × 3 × 1 Monkhorst–Pack mesh [64], and the cutoff energy for convergence was set to 500 eV. Oxygen‐terminating CeO_2_(111) was represented with a *p*(7 × 7 × 2) slab. Each slab was separated with a 20 Å vacuum layer along the *z*‐axis. Following the recommendations of previous benchmarking work on ceria [65], the rotationally invariant DFT + U scheme of Liechtenstein et al. [66, 67] was used to more accurately describe the electrons in the Ce 4f orbitals, with a Hubbard correction parameter *U*_eff_ of 4 eV (*U* = 5 eV, *J* = 1 eV). Infrared spectra were generated from the optimized structures using an in‐house code. The transition state associated with decarboxylation was identified using the improved dimer method [68, 69] as implemented in VASP.

## Conflicts of Interest

The authors declare no conflicts of interest.
